# An evaluation of posterior cruciate ligament reconstruction surgery

**DOI:** 10.1186/s12891-020-03533-6

**Published:** 2020-08-08

**Authors:** Mohammad Razi, Saman Ghaffari, Alireza Askari, Peyman Arasteh, Elaheh Ziaei Ziabari, Haleh Dadgostar

**Affiliations:** 1 Department of Orthopedic Surgery, Rasoul Akram Hospital, Iran University of Medical Sciences, Tehran, Iran; 2 Ziaeian Hospital, Tehran University of Medical Sciences, Tehran, Iran; 3 Bone and Joint Reconstruction Research Center, Shafa Orthopedic Hospital, Iran University of Medical Sciences, Tehran, Iran; 4 Department of Orthopedics, School of Medicine, Shiraz University of Medical Sciences, Shiraz, Iran; 5 Shiraz University of Medical Sciences, Shiraz, Iran; 6 Iran University of Science and Technology, Tehran, Iran; 7 Department of Sports Medicine, Rasoul Akram Hospital, Iran University of Medical Sciences, Tehran, Iran

**Keywords:** Posterior cruciate ligament, Reconstruction, Outcome, Iran, Trauma

## Abstract

**Background:**

The nature of posterior cruciate ligament (PCL) injuries and the scarcity of data on this issue have made reports on clinical and epidemiological features of PCL injuries valuable. We aimed to report our experiences with PCL injuries in our region.

**Methods:**

Any patient who referred with a diagnosis of PCL rupture from 2004 to 2018 to our center, was included in this report. We evaluated pre- and postoperative outcomes and compared patients with isolated and combined (multi-ligament) PCL injuries.

**Results:**

Overall, 55 patients were included in our study. Majority of patients were men (87.2%). Mean age of patients was 28.12 ± 8.53 years old.

Average follow-up period was 28.83 ± 20.62 months and mean duration between trauma and surgery was 27.8 ± 38.0 months. Most common cause of PCL injury was traffic accidents (70.9%) followed by sports injuries (5.5%). Majority of patients (69.1%) had combined PCL injuries.

Majority of patients underwent single tibial-double femoral tunnel reconstruction (56.4%), followed by single tibial-single femoral tunnel (34.5%) reconstruction. Allografts were used in 60% of patient. Average Cincinnati knee rating scale (CKRC) was 35.87 ± 11.4, which improved significantly after PCL reconstruction (79.45 ± 11.90, *p* <  0.001). Full range of motion only existed in 29.1% of patient prior to surgery, which improved after surgery (92.7%, *p* <  0.001).

Three patients had postoperative arthrofibrosis and motion stiffness, 1 had deep vein thrombosis and 3 patients had infections.

Those with isolated PCL injuries had higher pre-operative CKRS (42.05 ± 8.96 vs. 33.10 ± 11.45, *p* = 0.006) and lower pre-operative posterior drawer test (2.76 ± 0.43 vs. 3.1 ± 0.6, *p* = 0.042) compared to those with combined injuries.

**Conclusion:**

Today with advances in surgical techniques, considering treatment of collateral ligament injuries, use of stronger allografts and more secure fixation methods, better rehabilitation programs and early range of motion, results of reconstruction of the PCL has become very promising. Accordingly we recommend surgical treatment even for isolated PCL tears, with the goal to prevent functional deficit and to prevent degenerative arthritis.

## Background

Incidence of posterior cruciate ligament (PCL) injuries is variable. These injuries account for an estimated 1 to 47% of all acute knee ligament injuries [1–5] and 3% of all outpatient visits for knee injuries [4]. The PCL is among the strongest ligaments in the knee and is resistant to injuries and mechanisms that cause injuries to the PCL are not very common. Some of which include direct trauma to the anterior of the flexed knee (for example in dashboard injuries), hyper extension injuries, and severe varus or valgus injuries which usually cause multi-ligament injury [6].

Treatment of PCL injuries, mainly isolated PCL injuries, includes both surgical and non-surgical modalities, although some studies have advocated surgical treatment for isolated PCL injuries as well, data on this matter remains scarce [7, 8]. A recent study reported functional score to be significantly lower among individuals with isolated PCL injuries compared to those with isolated anterior cruciate ligament (ACL) injuries at time of reconstruction [7], yet reconstruction of the PCL makes up only 2–3% of cruciate ligament repairs [9].

The nature of PCL injuries and the scarcity of data on this issue have made reports on clinical and epidemiological features of PCL injuries valuable, furthermore studies significantly vary in their reports on clinical characteristics of PCL injuries [10]. Moreovere, as a result of the low incidence of PCL injuries, many studies on PCL reconstruction have been in the context of case series with little data on the management and long-term outcomes [11–13].

Data on injuries to the PCL mostly originate from Scandinavia and to the best of the authors knowledge, ligament registries are almost specific to this regions [10], whereas data from other parts of the world is widely missing.

Therefore, we sought to report clinical characteristics and treatment outcomes among our patients with PCL injuries.

## Methods

### Study design and settings

This is a descriptive study conducted in Hazrat Rasoul Hospital in Tehran, Iran affiliated to Iran University of Medical Sciences, Tehran, Iran. The center is a referral center for knee surgery for a population living in Tehran city and is among the few hospital in the country where PCL surgery is performed.

### Patients and study protocol

Any patient who referred with a diagnosis of acute or chronic tear of PCL with signs of functional deficit, pain, or instability of grade 2 or higher (based on the posterior drawer test) and had a displacement of more than 5 mm on stress x-ray, during 2004 up to 2018 was included in this report. Those who did not refer for their follow-up visits, individuals with concomitant knee deformity who underwent corrective osteotomy without any other reconstructive surgeries, and finally individuals with neuromuscular diseases or severe degenerative joint disease, were excluded from the study.

PCL tear was diagnosed based on physical examination and magnetic resonance imaging (MRI). Physical examination included posterior drawer test (PDT) and palpation of medial and lateral step-off, varus and valgus stress tests and evaluation of posteromedial and posterolateral corners. More specifically and especially in patients with isolated PCL tear, PDT was performed in acute flexion of the knee, during which palm of the examining hand was placed on the tibial tuberosity and anterior plateau and fingers of the same hand were placed on the patella and femoral condyles, while pushing the tibia back by the palm, posterior translation of the tibial plateau in relation to the femoral condyles was felt. The same process was performed on the uninvolved contralateral knee to determine the severity of the instability (supplement 1).

**Additional file 1.**

Among patients with grade 2+ to 3+ instability (in PDT), single PCL injury and no concomitant collateral ligament injury, a single tunnel PCL reconstruction of the anterolateral bundle was conducted. For more severe grade 3+ instability (in PDT) with substantial sagging of the knee, double tunnel PCL reconstruction was used. Depending on the size of the knee and based on the clinical assessment of the surgeon, double femoral tunnel - double tibial tunnel or double femoral tunnel - single tibial tunnel PCL reconstruction was used. For those who had posteromedial corner injuries a double tunnel PCL reconstruction was used with a posteromedial corner reconstruction. In patients with concomitant collateral ligament injuries, depending on the type of injury, double tunnel PCL and arthroscopic assisted tibialis posterior allograft MCL reconstruction or double tunnel PCL and arthroscopic popliteus tendon allograft reconstruction and the modified Larson technique was used.

### Surgical technique

Under spinal or general anesthesia, the involved limb was hanged from the end of the operating table and was prepared so that access to the posterior part of the knee and creation of the posteromedial (and if needed posterolateral) arthroscopy portal was feasible. Anterior working portals included high and low anterolateral portals and a high anteromedial portal.

**Fig. 1:**
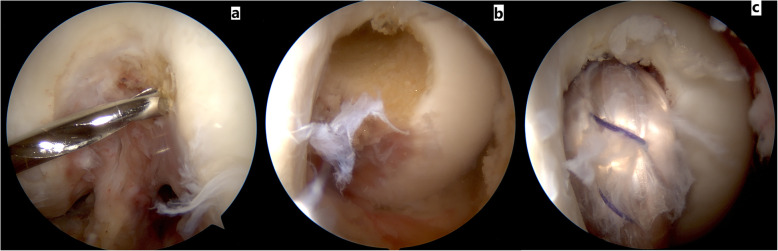
These images depict the single bundle PCL reconstruction technique. In figure **a** from a low anterolateral arthroscopic portal the aiming pin is directed towards the anterolateral bundle of PCL. Figure **b** shows a view of the anterolateral tunnel of PCL from a lateral portal. Figure **c** shows a view of the femoral attachment of single bundle PCL from a high anterolateral portal

**Fig. 2:**
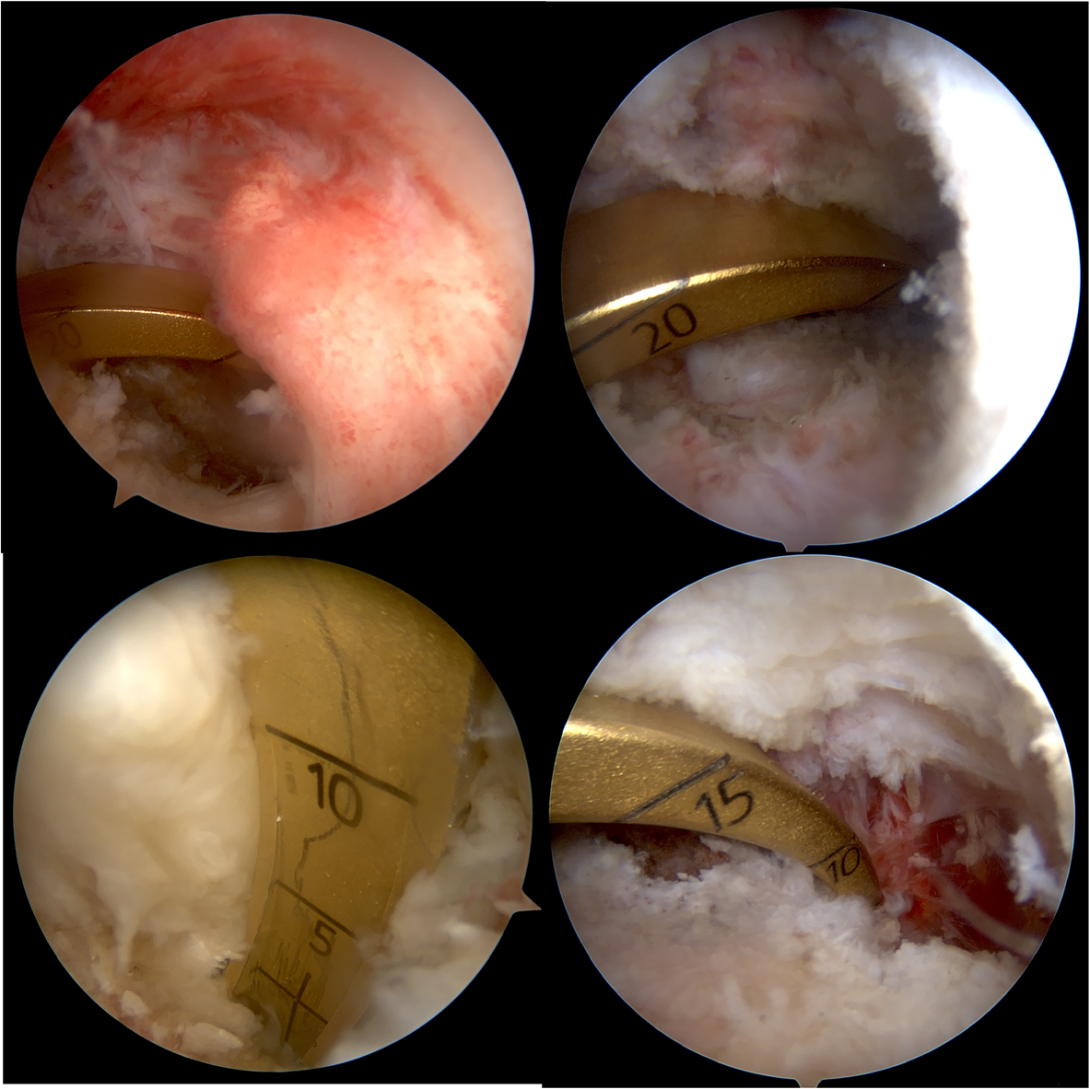
These images show that from high anteromedial arthroscopic portal, PCL jig is seated deeply enough to the posterior anatomic attachment of the PCL

For the single tibial-single femoral tunnel technique, first the femoral tunnel is created using a low anterolateral arthroscopic portal through which a guide pin is placed. Using a drill bit which has the same diameter as the looped tibialis posterior allograft tendon we then check that the footprint of the anterolateral bundle of the PCL is adjusted. Through the low anterolateral portal we pass the 30 degree scope into the posteromedial side of the popliteal fossa, adjacent to the remnant of the PCL, we enter into the space of the posteromedial corner and under a direct vision, the posteromedial portal is created. Care must be taken not to injure the saphenous nerve and vein and the popliteal neurovascular complex. Using a radiofrequency probe, through a posteromedial portal, under direct vision, from the posterior of the remnant of the tibial attachment of the PCL, while the face of the probe is towards the bone, dissection is done until the proximal border of the popliteus muscle is reached. After which, through the high anteromedial portal, jig of the PCL is entered into the popliteal space and under visual control by the arthroscopy scope through the posteromedial portal, the tip of the jig must sit on the anatomic tibial attachment site of the PCL. The angle of the jig is adjusted to 60 degrees. Tip of the newly commercially available PCL jigs are wide enough to stop and prevent extrusion of the tip of the guide pin into the popliteal fossa, especially if used under direct vision through the posteromedial arthroscopy portal, consequently there is no need to use other protective instruments such as arthroscopy curates or etc. Using a guide pin from the anteromedial part of the tibial plateau, medial to the tibial tuberosity we drill through the jig until the tip of the guide wire is visible in the correct position. It should be mentioned that with adequate vision much care should be given to protect the popliteal artery and vein. Especially for novice surgeons it is mandatory to check the position and direction of the guide wire by the C-Arm. After which using a drill bit, an appropriate tibial tunnel is created. Then the tibialis posterior allograft which is loaded with an endobutton is entered through the anteromedial arthroscopy portal of the knee. We can also enter the allograft through the tibial tunnel and then pass it into the joint and finally in the femoral tunnel. The head of the allograft which has the endobutton, is drawn from inside of the knee to the femoral tunnel and is then flipped over the medial femoral condyle. Considering that for allografts we used double fixation, in addition to an endobutton, a bio-interference screw is also placed inside the femoral tunnel. Thereafter, the force of the anterior drawer is applied into the proximal tibia until correction of the medial step-off in 90 to 70 degrees flexion of the knee, furthermore the tibial tunnel is fixed by one bio-interference screw and as double fixation the end of the tendon is also fixed to the tibial plateau by one tendon staple (Figs. 1 and 2).

**Fig. 3:**
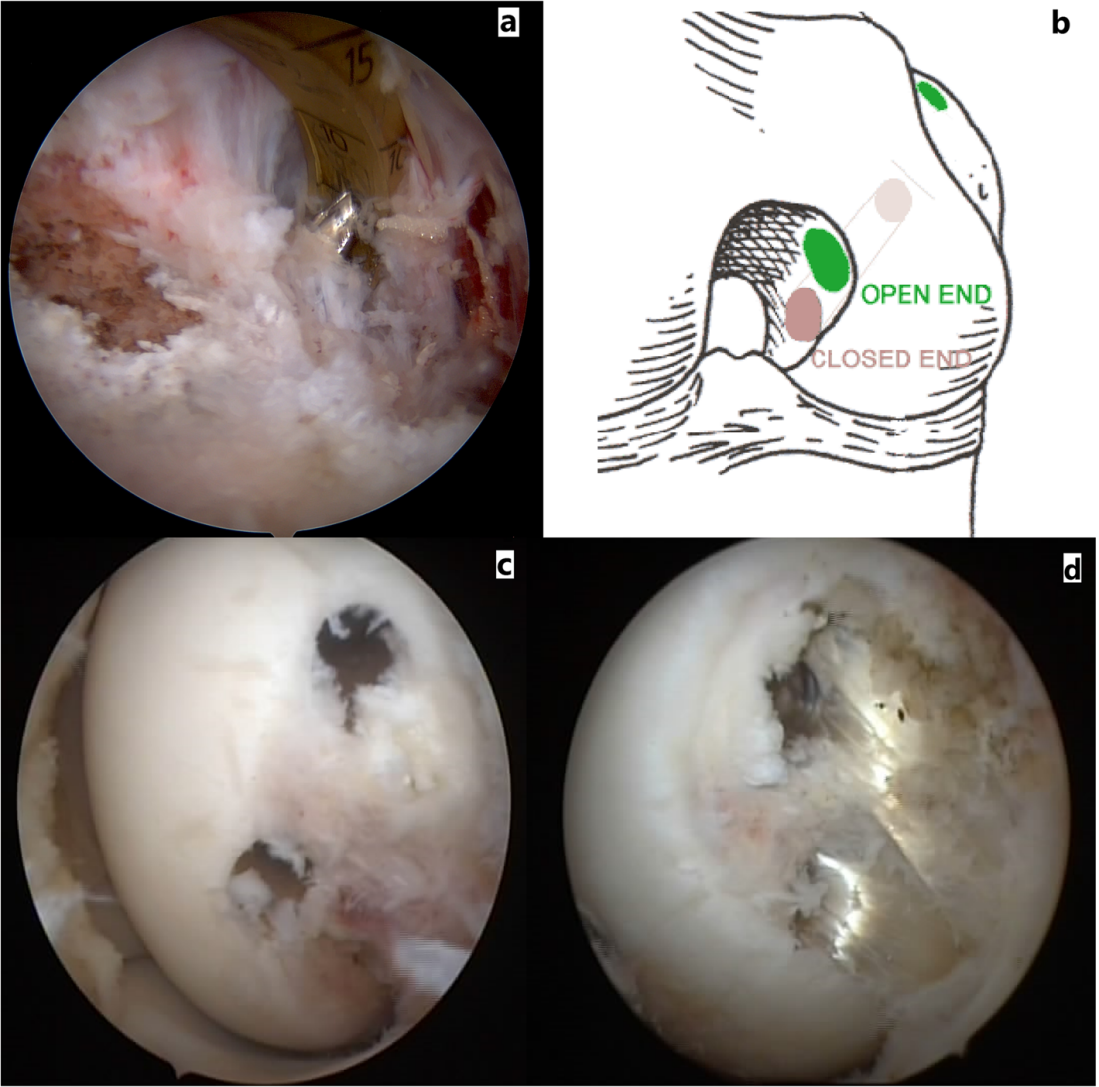
Figure **a** shows that from anteromedial plateau, the aiming pin is directed to the anatomic insertion of the PCL. Figure **b** shows a schematic view of femoral tunnels in double bundle PCL reconstruction. Figures **c** and d show arthroscopic views of femoral tunnels in double bundle PCL reconstruction

In the double femoral tunnel technique, two single strand sections of tibialis posterior allograft are prepared. Anterolateral femoral tunnel is created in the same way as in the single tunnel reconstruction technique and for the posteromedial tunnel, from the low anterolateral arthroscopy portal, a guide pin is inserted in the anatomic footprint of the posteromedial bundle of PCL just inferior to the meniscofemoral ligament during which care must be taken not to injure the meniscofemoral ligament, moreover a drill bit of appropriate size is used to create the tunnel. For the double femoral tunnel and single tibial tunnel PCL reconstruction, we create an open-ended anterolateral tunnel and a closed-ended posteromedial femoral tunnel (Fig. 3). In this way at the time of fixation, first we fix the femoral side of the posteromedial bundle by a bio-interference screw using the inside-out technique and then we put tension on all strands of the graft in almost knee extension and we fix both grafts in the tibial tunnel, after which we flex the knee and place tension on the anterolateral bundle (the open-ended tunnel) from the femoral side and finally we fix the femoral tunnel of the anterolateral bundle in 90 to 70 degrees of flexion using the outside-in technique. For augmentation fixations, we tie the non-absorbable sutures of the proximal end of the grafts over the medial femoral condyle and we augment the distal end of the grafts by one tendon staple on the medial tibial plateau just distal to the orifice of the tibial tunnel (Fig. 3).

For the double femoral - double tibial tunnel reconstruction, first the femoral tunnel of both bundles is fixed from inside of the joint by bio-interference screws in 90 degrees knee flexion then the tibial side of the posteromedial bundle is fixed in full knee extension and the tibial side of the anterolateral bundle is fixed in 70–90 flexion.

For PCL we only use tibialis posterior or tibialis anterior allografts.

### Follow-ups

For the first 6 months, follow-ups were different according to type of injury as either isolated PCL injury or combined injury, and based on the attending physician’s clinical assessment. Generally, after surgery patients were visited weekly for the first 2 weeks, after which for the next 3 months patients were followed every 3 weeks. After this period patients were given a follow-up at 6 months from their surgery which was then shifted to annual follow-ups. For evaluation of knee stability at follow-up visits, a bilateral kneeling stress radiography technique was used. This method of assessment was used to assess stability because of its easy applicability, faster response and relative low cost [14].

### Postoperative rehabilitation program and care

The first-week postoperative care included a simple knee immobilizer brace, no range of motion (ROM), partial weight bearing in the brace (in cases of no meniscus repair or collateral injury or reconstruction), isometric quadriceps exercises, and ankle pump.

In the second week, gentle passive and active ROM in the prone position was given and knee extension exercises were started.

After 2 weeks, straight leg raising was initiated and at the fourth week, depending on the condition of the quadriceps muscle, the knee immobilizer brace was removed. At 6 weeks, bilateral crutches were changed to a single crutch on the uninjured side.

### Definition of variables

Data on age, sex, job, time period between initial injury and surgery, type of trauma, chief complaint of patients, side of injury, type of injury as either single PCL injury or combined injury, type of PCL surgery, type of graft used for surgery, period of follow-up, pre- and PDT grade, Cincinnati knee rating scale (CKRS) before and after surgery, range of motion (ROM) before and after surgery, and complications, were recorded for each patient.

Type of trauma was categorized as traffic accidents, sports related injuries, and other types of injuries (falling down and etc.) which were classified as miscellaneous.

Patients’ chief complaints were either instability or instability and pain of the knee.

Type of PCL surgery was classified as single tibial - single femoral tunnel, single tibial - double femoral tunnel, and double tibial - double femoral tunnel.

Type of graft used was either autograft or allograft.

Surgery related complications were categorized as infections, arthrofibrosis and motion stiffness, and deep venous thrombosis.

Associated injuries included medial and lateral meniscal injuries, chondral lesions, ACL tear, popliteus and patellar tendon, posteromedial and posterolateral corner injuries.

In order to measure surgery related outcomes, the CKRS [15] was used. Multiple outcome measuring systems have been introduced, however only two have had acceptable validity and reliability, one of which is the CKRS. This measure includes indexes such as swelling of the knee, pain, giving away, overall daily activity level, walking, use of stairs, running activity, and jumping and twisting. The index has been shown to have high test reliability and validity in ACL injuries and has been also used in the context of PCL injuries [16, 17]. The index was measured 1 day before surgery and at final follow-up.

Using the kneeling stress x-ray, posterior displacement was measured and compared to the contralateral uninvolved knee. For obtaining the stress x-ray, the patient was asked to bend their knee at 90 degrees and to put their whole weight on their tibial tubercle. For the measurement and calculating the amount of displacement, one point was defined along the posterior cortex 10 cm from the joint line distal to the tibial plateau. Following which a line was drawn from this point parallel to the posterior cortex directed toward the knee joint. The most posterior point of the Blumensaat line was also marked. Finally a perpendicular line from the Blumensaat line was drawn to intersect the first line. Accordingly, the distance was measured and reported as the amount of displacement.

### Ethical consideration

All patients’ personal data were secure throughout the study in order to protect anonymity of the patients. All patients gave their written and informed consent to enter the study. Study protocol has been approved by the Institutional Review Board of Fasa University of Medical Sciences (Ethics code #IR.FUMS.REC.1396.272).

### Statistical analysis

Data was analyzed using the SPSS® software for windows®, version 20 (SPSS Inc., Chicago, IL, USA). For comparison of quantitative variables with normal distribution between two groups (those with isolated PCL injuries and those with complex PCL injuries), the independent T-test and for comparison of qualitative variables between groups the Chi-square and Fisher’s exact test was utilized. For comparison of quantitative variables without normal distribution between two groups the Mann–Whitney test was used. For comparison of pre- and post-operative results in a single group the paired T-test was used. A *p*-value of less than 0.05 was considered statistically significant.

## Results

Initially, 68 patients were considered for the study. A total of 3 patients did not refer for their follow-ups, 8 cases had knee deformities and 2 patient had neuromuscular diseases and were excluded from the study. A total of 55 individuals entered our study.

Majority of patients were males (87.25%). Mean (SD) age of patients was 28.12 ± 8.53 years old, ranging from 17 to 53 years old. In total, majority of patients (69.1%) had combined (multi-ligament) PCL injuries. Other clinical characteristics are mentioned in Table 1.

**Table 1:** Baseline characteristics of the study population^a^

Variables	Statistics
Sex - no. (%)	
Male	48 (87.2)
Female	7 (12.7)
Age - yrs	28.1 ± 8.5
Duration of injury to surgery - months	27.8 ± 38.0
Cause of trauma - no. (%)	
Traffic accident	39 (70.9)
Sports related	3 (5.5)
Miscellaneous	13 (23.6)
Chief complaint - no. (%)	
Instability	47 (85.5)
Instability + Pain	8 (14.5)
Injury side - no. (%)	
Right	39 (56.4)
Left	24 (43.6)
Type of PCL injury - no. (%)	
Isolated PCL	17 (30.9)
Complex injury	38 (69.1)
Meniscal injuries - no. (%)	
Medial meniscus	5 (9.1)
Lateral meniscus	5 (9.1)
Both	2 (3.6)
No	43 (78.2)
Ligament injury - no. (%)	
ACL	4 (7)
ACL + posteromedial corner	3 (5.4)
ACL + posterolateral corner	4 (7)
ACL + posteromedial + posterolateral	1 (1.8)
Posteromedial corner	11 (20)
Patellar tendon + posteromedial	2 (3.6)
Posterolateral corner	12 (21.8)
Posterolateral corner + posteromedial corner	1 (1.8)
isolated	17 (30.9)
Chondral lesion - no. (%)	
Yes	17 (30.9)
No	38 (69.1)

*Abbreviation*: *PCL* posterior cruciate ligament

^a^Since we had a population of less than 100 individuals, all percentages have been rounded to the first decimal. All plus-minus values are means and standard deviations unless stated otherwise

Average follow-up period was 28.83 ± 20.62 months. Regarding type of PCL surgery, majority of patients underwent single tibial - double femoral tunnel surgery (56.4%), followed by single tibial - single femoral tunnel (34.5%) and double tibial - double femoral tunnel surgery (9.1%).

Regarding injury characteristics, preoperative PDT was 2+ in 16.4% of patients, 3+ in 67.3% of patients and 4+ in 16.4% of patients. Postoperative PDT improved in majority of patients (*p* <  0.001), as 74.5% had negative PDT with normal medial step-off, and the rest had a score of 1+ at final follow-up.

Average CKRS was 35.87 ± 11.4 prior to surgery, which significantly improved after PCL surgery at final follow-up (79.45 ± 11.90, *p* <  0.001).

Moreover, full ROM after PCL surgery at final follow-up was achieved in 92.7% of patients.

A total of 48 patients had no postoperative complications. Three patients had arthrofibrosis and motion stiffness, one had deep vein thrombosis and three patients had infections. Those with arthrofibrosis underwent arthroscopic arthrofibrolysis and full ROM was obtained at follow-up visits. The patient with deep vein thrombosis was hospitalized and treated with appropriate anticoagulant therapy. The three patients with infections received adequate antibiotic treatment and underwent arthroscopic lavage. No failures leading to reoperation was recorded with any of our patients. In follow-up radiography, overall 17 patients had a posterior tibial displacement of between 0 and 5 mm (Table 2).

**Table 2:** Clinical and treatment related characteristics of PCL injuries^a^

Variables	Statistics	***p***-value
Follow-up - months	28.8 ± 20.6	
Type of operation - no. (%)		
Single tibial - single femoral tunnel	19 (34.5)	
Single tibial - double femoral tunnel	31 (56.4)	
Double tibial - double femoral tunnel	5 (9.1)	
Type of graft - no. (%)		
Allograft	33 (60)	
Autograft	22 (40)	
PDT - no. (%)		
Preoperative		
2+	9 (16.4)	< 0.001
3+	37 (67.3)	
4+	9 (16.4)	
Postoperative		
0 or negative	38 (73.1)	
1+	14 (26.9)	
Cincinnati knee score		
Preoperative	35.9 ± 11.4	< 0.001
Postoperative	79.4 ± 11.9	
Full ROM - no. (%)		
Preoperative	16 (29.1)	
Postoperative	51 (92.7)	
Complications - no. (%)		
No	48 (87.3)	
Arthrofibrosis and motion stiffness	3 (5.5)	
Deep vein thrombosis	1 (1.8)	
Infection	3 (5.5)	

*Abbreviations*: *PCL* posterior cruciate ligament, *PDT* posterior drawer test, *ROM* range of motion

^a^All plus-minus values are means and standard deviations unless stated otherwise

Three patients developed postoperative arthrofibrosis and thus were not included in the assessment of postoperative PDT

When comparing those with isolated PCL injuries and those with combined PCL injuries, we found that those with isolated PCL injuries had longer follow-ups (*p* = 0.032), higher CKRS prior to surgery (*p* = 0.006), and lower pre-operative PDT (*p* = 0.042) (Table 3).

**Table 3:** Comparison of baseline and clinical data between PCl patients based on their type of PCL injury^a^

Variables	Type of PCL lesion		
	Isolated	Combined	*p*-value
Sex - no. (%)			
Male	17 (100)	31 (81.6)	0.058
Female	0	7 (18.4)	
Age - yrs	25.6 ± 8.4	27.9 ± 8.6	0.366
Duration of injury to surgery - months			
Median and IQR	28.8 ± 50.6	27.4 ± 31.6	0.970
	6 (3.75, 19)	19 (6, 39)	
Follow-up - months	37.7 ± 32.3	24.9 **±** 10.9	0.032
Cause of trauma - no. (%)			
Traffic accident	10 (58.8)	29 (76.3)	0.381
Sports related	6 (35.3)	7 (18.4)	
Miscellaneous	1 (5.9)	2 (5.3)	
Chief complaint - no. (%)			
Instability	15 (88.2)	32 (84.2)	0.696
Pain	2 (11.8)	6 (15.8)	
Injury side - no. (%)			
Right	5 (29.4)	19 (50)	0.155
Left	12 (70.6)	19 (50)	
Type of operation - no. (%)			
Single tibial - single femoral tunnel	3 (17.6)	16 (42.1)	0.115
Single tibial - double femoral tunnel	11 (64.7)	20 (52.6)	
Double tibial - double femoral tunnel	3 (17.6)	2 (5.3)	
Meniscal injury - no. (%)			
Medial meniscus	3 (17.6)	2 (5.3)	0.467
Lateral meniscus	1 (5.9)	4 (10.5)	
Both	1 (5.9)	1 (2.6)	
None	12 (70.6)	31 (81.6)	
Chondral lesion - no. (%)			
Yes	6 (35.3)	11 (28.9)	0.638
No	11 (64.7)	27 (71.1)	
Type of graft - no. (%)			
Allograft	10 (58.8)	23 (60.5)	0.905
Autograft	7 (41.2)	15 (39.5)	
PDT			
Preoperative	2.8 ± 0.4	3.1 ± 0.6	0.042
Postoperative	0.2 ± 0.4	0.3 ± 0.4	0.830
Cincinnati knee score			
Preoperative	42.0 ± 8.9	33.1 ± 11.4	0.006
Postoperative	83.8 ± 10.5	77.5 ± 12.1	0.068
Full ROM - no. (%)			
Preoperative	8 (47.1)	8 (21.1)	0.50
Postoperative	16 (94.1)	35 (92.1)	0.791
Complications - no. (%)			
No	16 (94.1)	32 (84.2)	0.587
Arthrofibrosis and motion stiffness	0	3 (7.9)	
Deep vein thrombosis	0	1 (2.6)	
Infection	1 (5.9)	2 (5.3)	

*Abbreviations*: *PCL* posterior cruciate ligament, *PDT* posterior drawer test, *ROM* range of motion, *IQR* interquartile range

^a^All plus-minus values are means and standard deviations unless stated otherwise. Values have been rounded

## Discussion

Inhere we reported on the clinical outcomes of patients undergoing PCL reconstruction from Iran. Due to the nature of the injury, in most countries only a few surgeon are experts in PCL reconstruction surgery, thus making reports on PCL injuries valuable.

To the best of the authors’ knowledge, three registries exist on knee ligament injuries, one of which, specifically focuses on PCL injuries. All these registries are in the Scandinavian region and include the Norwiegen, Swedish and the Danish registries [18–20].

The Norwegian National Knee Ligament Registry (NKLR), reported by Aroen et al. in 2012 [20] during 2004–2010, included 295 PCL injuries. The rate of isolated PCL injury in their study was lower compared to that of ours (24% vs. 30.9%). They found that sports injuries were the most common causes of isolated PCL injuries. In comparison to our series, we had a higher mean age, and higher duration between injury and surgery, furthermore majority of our patients were males, and majority of our injuries were caused by traffic accident (70%) rather than sports related injuries.

The Danish Knee Ligament Reconstruction Registry (DKRR) [21] started in 2005. They had a relatively large population with PCL injuries, which included 237 individuals with isolated PCL injury and 344 patients with combined PCL injuries. Their isolated PCL and combined PCL injury groups had a mean age of 31.8 ± 11.1 years and 33 ± 11.1 years, and rate of male patients of 69% and 73%, respectively. Majority of their injuries were caused by sports accidents (43%) and most of their patient received autograft reconstructions.

The Swedish ligament registry was initiated in 2005 and used a web-based protocol. The main goal of this registry focused on ACL injuries [19]. In a recent review by Owesen et al. [10] all Scandinavian registries on ligament injuries were assessed. They found that the mean age of patients with PCL injuries was 32.7 years, with men constituting the majority of patients (two thirds), and sports injuries, specifically football (soccer) to be the most common cause of injury.

Perhaps among the most important reasons why males constituted the majority of our patients, relates to the cause of injury, as in our study majority of injuries were caused by accidents which usually includes male drivers in our country, however in the Scandinavian region sports injuries were the most common cause of PCL injuries. Perhaps this could also be the reason for the lower age recorded in their study for PCL injuries, as younger individuals are involved on sports activities.

The reason we had a different cause for PCL injuries in our study compared to that of the Scandinavian region, maybe due to the fact that Iran has one of the highest rates of traffic accidents in the world [22], which predisposes individuals to traumatic injuries (including ligament injuries).

A total of four patients lacked full ROM after surgery. All these individuals had multi-ligament injuries and although they did not have a full ROM prior to surgery they all had improved ROM after PCL reconstruction.

We found that 30.9% of all PCL injuries were isolated PCL injuries and the rest were combined injuries. This was significantly lower than that reported by Schulz et al. [3], that found 47% of all PCL injuries to be isolated PCL injuries, furthermore our rates of isolated PCL injuries were higher than that reported by Fanelli et al. [4] who found a prevalence of 7.5% for isolated PCL injuries. This demonstrates a wide discrepancy between studies on epidemiologic features related to PCL injuries, which can be due to the rarity of the condition.

One of the reasons for a longer period between injury and operation for those with isolated PCL injuries, is the longer time period in which isolated PCL injuries are allowed to recover before surgical intervention is considered, as some individuals with isolated PCL injuries seek non-surgical treatments as opposed to combined PCL injuries in which surgical treatment is considered the primary treatment approach [3].

From another aspect non-surgical or conservative treatment of isolated PCL injuries have been advocated by some authors. One of the largest series included 68 patients with isolated PCL injuries who were treated using conservative measures and were followed for ten years. in this study Shelbourne et al. [16] reported that during the long term follow-up, all of their patients regained full ROM, furthermore patients’ quadriceps strength was almost similar to the uninjured leg. On the other hand, 11% of their patients developed moderate to severe degrees of osteoarthritis. More importantly as conservative treatment of isolated PCL injuries are usually considered for patients with low grade PCL injuries [23], a cross comparison of outcomes with that of surgical treatment is difficult.

Two of the three patients who developed postoperative infections in our study, had multi-ligament injuries (one had ACL tear and the other had ACL plus posteromedial and posterolateral corner injuries). Furthermore, those who developed postoperative arthrofibrosis in our study all had multi-ligament injuries. From another aspect the relative high rate of infections (5.5%) and arthrofibrosis (5.5%) in our report could also be attributed to the low sample size of the study.

The study results allow a comprehensive assessment of patient characteristics, surgery specifics, benefits and complications related to PCL surgery in our region, which is a relatively uncommon condition in orthopedic surgery. Moreover, in most instances of trauma to the knee the ACL is injured and the PCL is spared, so reports on PCL injuries render valuable information regarding treatment and clinical course of the disease and will aid in the correct management of the condition.

One interesting point in our study included the use of allografts for PCL reconstruction, which is not accessible in many countries. We found excellent results with allograft reconstructions, moreover it should be mentioned that actually no suitable and harvestable autograft exists for PCL reconstruction, especially in multi-ligament injuries.

This is among the first studies on PCL to use a functional questionnaire to assess postoperative outcomes using the CKRS. Borsa et al. [24] evaluated individuals with ACL injuries and aimed to evaluate the effectiveness of performance-based or patient-reported measures of function and disability. They used subjective ratings of knee function as the criterion for disability. They found that the CKRS was among the three most effective estimators of disability.

This study was not without limitation. This was a report from a single center and although this was among the main referral centers for PCL surgery, findings of the study should be interpreted with caution. The hospital in which the study was conducted performs 5 cases of PCL surgeries per year which is higher than most hospital reports, as some hospitals in Norway and Sweden are reported to perform as low as one case of PCL reconstructive surgery each year [10]. All scoring of patients and related examinations were performed by the supervising attending surgeon both at initial assessment and during follow-ups, minimizing the between-observer variability seen in previous literature.

## Conclusions

In the past, different recommendations existed on the conservative treatment of isolated PCL injuries in literature and perhaps due to the fact that after PCL reconstruction, degrees of limitation in ROM and instability would remain and in some instances relapsing of the instability would occur, moreover complications such as popliteal neurovascular injuries would make the surgeon reluctant to perform reconstructive surgery. Today with advances in surgical techniques, considering treatment of collateral ligament injuries, use of stronger allografts and more secure fixation methods, better rehabilitation programs and early ROM, results of reconstruction of the PCL has become very promising. Accordingly we recommend surgical treatment even for isolated PCL tears, with the goal to prevent functional deficit and to prevent degenerative arthritis.

## Data Availability

All authors and institutions can request data related to the study from the primary supervising author at Mrazi_md@yahoo.com.

## Abbreviations

PCL: Posterior cruciate ligament
CKRC: Cincinnati knee rating scale
PDT: Posterior drawer test
ROM: Range of motion
